# Invasion Mechanisms of the Alien Plant *Datura stramonium* in Xizang: Insights from Genetic Differentiation, Allelopathy, and Ecological Niche Analysis

**DOI:** 10.3390/biology14111629

**Published:** 2025-11-20

**Authors:** Yonghao Chen, Zhefei Zeng, Qiong La, Junwei Wang

**Affiliations:** Key Laboratory of Biodiversity and Environment on the Qinghai-Tibetan Plateau, Ministry of Education, School of Ecology and Environment, Xizang University, Lhasa 850000, China

**Keywords:** invasive plant, *Datura stramonium*, genetic differentiation, allelopathy effect, niche characterisation

## Abstract

*Datura stramonium*, which is originally native to Mexico, has been recognized as an invasive species following its introduction to China, where it has proliferated extensively. Despite its widespread impact, the mechanisms driving the invasion of *D. stramonium* remain insufficiently understood. Therefore, gaining insight into these mechanisms is essential for the development of effective strategies to prevent and control its further invasion. This study aims to elucidate the factors contributing to the successful invasion of *D. stramonium* in Tibet by examining genetic differentiation, allelopathic potential, and niche characteristics of its populations. Our findings reveal the following: (1) The genetic variation within 15 populations of *D. stramonium* is predominantly intra-populational, lacking distinct genealogical phylogeographic structure, and is indicative of recent population expansion. This suggests that human-mediated dispersal has played a significant role in the invasion of *D. stramonium* in Tibet. (2) Allelopathic assays demonstrate that extracts from various parts of *D. stramonium* exhibit significant inhibitory effects on the germination of *Hordeum vulgare* var. *coeleste* and *Pisum sativum* seeds. (3) *D. stramonium* exhibits the highest niche breadth within the plant community, coupled with a pronounced competitive ability for environmental resources. The invasion of *D. stramonium* poses a substantial threat to the diversity of local plant species. Consequently, the formulation of scientific management measures is of critical importance to prevent and control the invasion of *D. stramonium* and preserve biodiversity in the invaded area.

## Simple Summary

*Datura stramonium*, a poisonous plant originally from Mexico, is spreading quickly in parts of China, including Tibet, and can crowd out local plants. We aim to understand why it is so successful so that better control plans can be made. First, by studying differences in plant DNA across 15 sites, we found that most differences occur within each site rather than between sites, and the overall pattern looks recent. This indicates that people and transport help the plant move and establish new populations. Second, tests using liquids extracted from the plant’s leaves, stems, and roots showed that these extracts strongly slowed or stopped seed sprouting in barley and pea, which means that *D. stramonium* can chemically suppress neighbors. Third, when we compared how widely the species use space and resources in local plant communities, *D. stramonium* had the broadest “niche,” indicating that it competes very effectively for light, water, and nutrients. Together, these findings show that *D. stramonium* threatens local biodiversity in Tibet. Early detection, limits on unintentional transport, and targeted removal are needed to protect native plants and the services they provide to people.

## 1. Introduction

*Datura stramonium* L. is an annual herbaceous plant belonging to the genus Datura within the Solanaceae family. It is widely distributed across temperate and subtropical regions, often thriving in disturbed areas such as waste grounds and gardens [1]. Although native to Mexico, historical records such as the *Compendium of Materia Medica* (1578) indicate that *D. stramonium* was introduced to China as a medicinal plant. However, the absence of long-term normative regulatory measures has led to its extensive proliferation across various provinces and cities in China [2], where it is now classified as an invasive species [3]. While the species has been present in China for several centuries, its occurrence in Tibet has only been documented in recent decades, and it is now recognized as an invasive species in this region. *D. stramonium* is known for its potent allelopathic effects, which can significantly inhibit the germination and growth of economically important crops [4], thereby impacting the biodiversity of the ecosystems it invades [5]. The mechanisms underlying plant invasions are complex and multifaceted, involving a range of factors such as the allelopathic properties of the invasive plant [6,7,8], its environmental adaptability [9], and anthropogenic influences [10]. Several hypotheses have been proposed to explain the success of invasive species, including the “inherent superiority hypothesis” [11], which attributes invasiveness to the biological traits and competitive advantages of the exotic species itself; the “evolution of increased competitive ability hypothesis” [12], which focuses on the interactions between invasive and indigenous species; the “novel weapons hypothesis” [13]; the “diversity resistance hypothesis” [14]; “empty niche hypothesis” [15] for the invasiveness of new habitat environment, and the “distraction hypothesis” [16]. Nonetheless, these hypotheses are often insufficient on their own, as the invasion process frequently involves multiple interacting factors.

The invasion mechanism of *D. stramonium* remains largely unexplored, and no comprehensive studies combining various methodological approaches to analyze this phenomenon from different perspectives have been conducted. It is crucial to investigate the factors contributing to the successful invasion of *D. stramonium* from different perspectives, including genetic differentiation, allelopathic effects, and ecological niche characteristics. To this end, we employed a combination of methods, including chloroplast non-coding regions sequencing, Petri dish filter paper assays, and sample survey method, to elucidate the underlying invasion mechanisms of *D. stramonium*.

## 2. Materials and Methods

### 2.1. Materials and Experimental Methods for Assessing the Allelopathic Effects of D. stramonium

Plant materials of *D. stramonium* used for allelopathic assays were collected in the summer of 2023 from Lhasa City, Tibet Autonomous Region, China. To avoid potential bias caused by single-plant effects, tissues were sampled from 15 different healthy individuals. All collected plants were cleaned, naturally air-dried, and then separated into roots, stems, and leaves. The dried tissues from the 15 individuals were pooled and homogenized together, ground into fine powder, and finally passed through a 40-mesh sieve.

For allelopathic treatments, 0.5 g, 1.0 g, 1.5 g, and 2.0 g of the mixed powders were weighed and placed into sterilized Petri dishes lined with double-layer filter paper; a blank control was included. Each dish received 20 mL of distilled water and was allowed to soak at 25 °C for 24 h. Seeds of *Hordeum vulgare* var. *coeleste* and *Pisum sativum*, selected for uniformity and good condition, were surface-sterilized in sodium hypochlorite solution, rinsed with sterile water, and evenly placed in the prepared Petri dishes. Each treatment consisted of three replicates. The dishes were incubated in a light-controlled growth chamber at 25 °C, with a 12 h/12 h light–dark cycle (light intensity level 3). Distilled water was added as needed to maintain adequate moisture during germination.

### 2.2. Molecular Materials and Experimental Methods for D. stramonium

**Figure 1:**
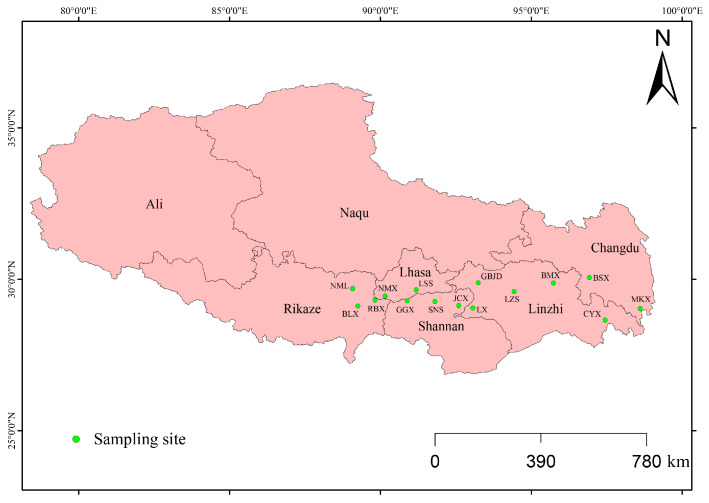
Location map of 15 *D. stramonium* populations.

Molecular samples of *D. stramonium* were collected from 15 populations distributed across multiple cities and counties in Tibet, China (Figure 1; detailed information in Table 1). All sampled plants were taxonomically identified by Dr. Junwei Wang of Xizang University prior to collection. For each population, at least 20 individuals were sampled, resulting in a total of 336 individuals. To reduce spatial autocorrelation, plants were collected at intervals of more than 50 m. For each population, one voucher specimen was collected and deposited in the Herbarium of the College of Ecology and Environment, Tibet University, with voucher numbers Wjw20230701–Wjw20230715.

Genomic DNA was extracted using the TIANGEN DP321 kit (TIANGEN BIOTECH Co., Ltd., Beijing, China), and DNA quality was examined by 1% agarose gel electrophoresis. Polymerase chain reaction (PCR) amplification was performed using the chloroplast trnL fragment as a molecular marker [17], with the forward primer F-CGAAATCGGTAGACGCTACG and the reverse primer R-GGGGATAGAGAGGGACTTGAAC. Each 50 μL PCR contained 25 μL Vazyme Taq PCR MasterMix (Vazyme Biotech Co., Nanjing, China), approximately 20 ng of genomic DNA, 0.2 μM of each primer, and nuclease-free water to a final volume of 50 μL. The PCR program included an initial denaturation at 94 °C for 3 min; 30 cycles of 94 °C for 30 s, 50 °C for 30 s, and 72 °C for 1 min; followed by a final extension at 72 °C for 5 min. Amplification products were verified by 1% agarose gel electrophoresis and subsequently sequenced at the Beijing Genomics Institute (BGI).

### 2.3. Experimental Methods for Analyzing D. stramonium Communities

To assess the community composition and plant diversity associated with *D. stramonium*, a total of 26 sample plots (3 m × 3 m) were established in areas surrounding Lhasa City, Tibet Autonomous Region. Lhasa was selected as the survey region due to its high level of human disturbance, which provides suitable conditions for examining the invasion patterns and community impacts of *D. stramonium*.

Within each plot, all co-occurring plant taxa were recorded, including their scientific names, number of individuals, and percent cover. Species identification was conducted in the field by Dr. Junwei Wang (Xizang University). For taxa that could not be reliably identified on-site, specimens were pressed and transported to the laboratory for further verification using the Flora of China Online (http://www.iplant.cn/) and the Chinese Virtual Herbarium (CVH, https://www.cvh.ac.cn/). All collected specimens were deposited in the Herbarium of the College of Ecology and Environment, Tibet University, under the collection code MTLYF-2023.

### 2.4. Data Analysis

Seed germination was defined as the radicle breaking through the seed coat by 1–2 mm. The number of germinated seeds was recorded daily until no further changes were observed. Germination percentage, germination potential, germination index, and allelopathic inhibition were calculated according to standard methods [18]. The overall allelopathic inhibition effect of *D. stramonium* on the germination of *H. vulgare* var. *celeste* and *P. sativum* seeds was evaluated in a composite manner by calculating the arithmetic mean of the inhibition rates from multiple determinations of the donor on the same acceptor relative to the control. Statistical analyses were performed using SPSS 27.0, and graphs were generated using Origin 2021.

Sequence comparisons were performed using MEGA 7.0.26, while haplotype analysis and neutrality tests (Tajima’s D test) were conducted using DnaSP 5.10.01. Haplotype networks were constructed using Network 10.2, and mismatch and analysis of molecular variance (AMOVA) analyses were performed using Arlequin 3.5.1.2. Finally, the haplotype diversity index (Hd) of *D. stramonium* populations was correlated with environmental factors using R 4.3.1.

The importance value of each species in the sample, community species diversity index [19,20], invasion intensity index [21], species niche width [22,23], and species niche overlap [24] were calculated. The correlation between the community species diversity index and the invasion intensity index was analyzed using R 4.3.1.

## 3. Results

### 3.1. Allelopathic Effects of D. stramonium

**Figure 2:**
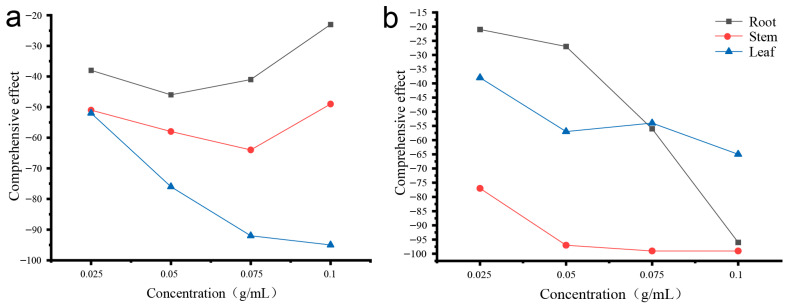
Comprehensive effects of aqueous extracts of *D. stramonium* roots, stems, and leaves on seed germination of two recipient species: (**a**) *H. vulgare* var. *coeleste*; (**b**) *P. sativum*.

The comprehensive allelopathic effects of aqueous extracts from *D. stramonium* roots, stems, and leaves on seed germination of the two tested species are presented in Figure 2. For *H. vulgare* var. *coeleste* (Figure 2a), leaf extracts consistently showed the strongest inhibition across all concentrations, with the most pronounced reduction at 0.075–0.1 g/mL. Stem extracts caused moderate inhibition, whereas root extracts exhibited the weakest overall effect. For both root and stem extracts, the comprehensive inhibition values at 0.075–0.1 g/mL were slightly less negative than those observed at 0.05 g/mL. This pattern reflects the non-linear responses of the three germination parameters—germination rate, germination potential, and germination index—to increasing extract concentrations, resulting in a higher mean value at the highest concentrations.

For *P. sativum* (Figure 2b), stem extracts produced the strongest suppression, showing a sharp decline in comprehensive effect with increasing concentration and reaching the lowest value at 0.1 g/mL. Root extracts produced a similar concentration-dependent decline, with the largest inhibition also occurring at 0.1 g/mL. Leaf extracts showed moderate inhibition, with relatively smaller variation among concentrations. As in the previous analysis, the comprehensive allelopathic effects reflect the arithmetic mean of the three germination parameters, which explains the slight fluctuations observed among different concentrations.

### 3.2. Population Differentiation, Demographic History, and Haplotype Distribution of D. stramonium in Tibet

#### 3.2.1. Genetic Differentiation and Historical Demographic Patterns of *D. stramonium*

Genetic differentiation among the 15 *D. stramonium* populations in Tibet was low, with an overall Fst value of 0.05871, indicating weak genetic structure across regions. The inter-population differentiation indices Nst (0.05782) and Gst (0.09461) also exhibited low levels of divergence, and the observation that Nst < Gst suggests an absence of clear phylogeographic structure among populations.

**Figure 3:**
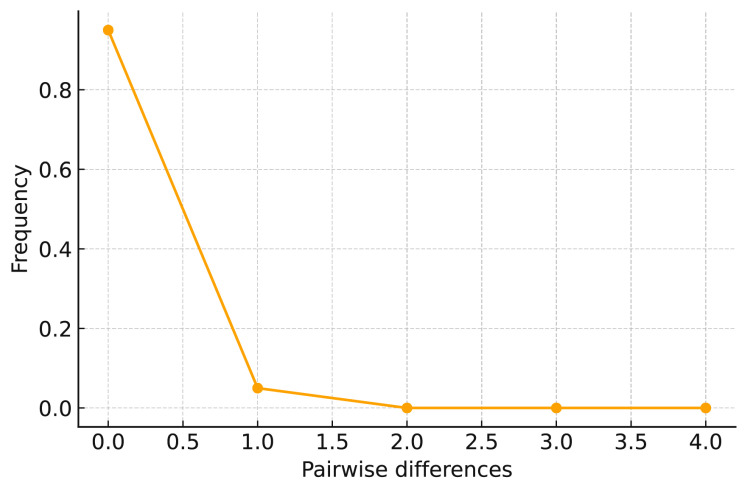
Mismatch analysis of 15 *D. stramonium* populations in Tibet, China.

Historical demographic analysis revealed signals of recent population expansion. Tajima’s D was significantly negative (D = −1.76007, *p* < 0.05), indicating an excess of low-frequency polymorphisms. The mismatch distribution curve (Figure 3) displayed a unimodal pattern with a steep initial decline followed by a gradual tail, which is characteristic of populations that have experienced sudden demographic growth. These combined results suggest that *D. stramonium* populations in Tibet exhibit weak genetic differentiation and have undergone recent expansion.

#### 3.2.2. Haplotype Distribution in *D. stramonium* Populations

**Figure 4:**
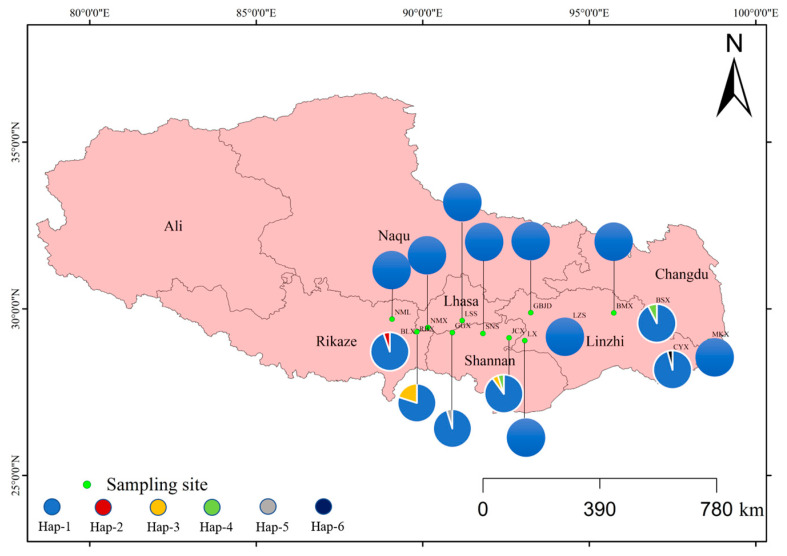
Geographical distribution of haplotype of 15 *D. stramonium* populations in Tibet, China. Note: The acronyms stand for the following regions: Linzhi city (LZS), Renbu county (RBX), Gongbujiangda county (GBJD), Lang county (LX), Bailang county (BLX), Lhasa city (LSS), Bomi county (BMX), Chayu county (CYX), Basu county (BSX), Gongga county (GGX), Nanmulin county (NML), Mangkang county (MKX), Nimu county (NMX), Jiacha county (JCX), Shannan city (SNS).

Six haplotypes were identified among the 15 *D. stramonium* populations in Tibet, with their geographical distributions depicted in Figure 4. Hap-1 was present in all 15 regions, while Hap-2, Hap-5, and Hap-6 were private haplotypes found only in specific regions. Hap-3 and Hap-4 were observed in two regions, though these regions were geographically distant from each other.

**Figure 5:**
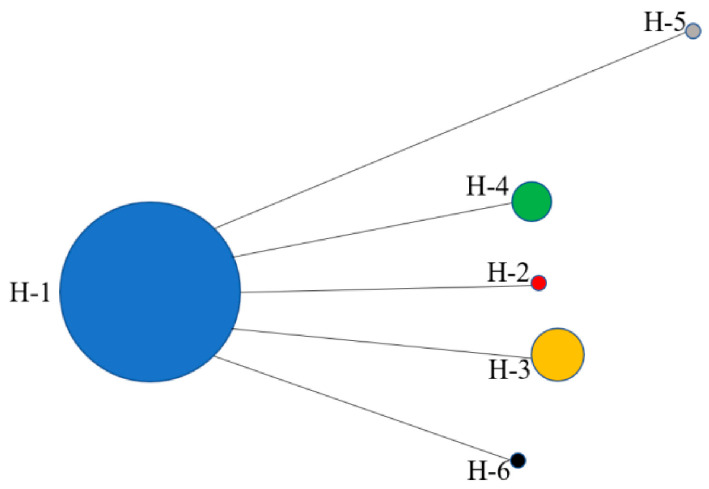
Median-Joining network of 6 haplotype of *D. stramonium* populations of Tibet, China.

The mediator network diagrams for the six haplotypes (Figure 5) reveal that Hap-1 is positioned on the trunk of the mediator network diagram, suggesting that it was differentiated earlier and is the original ancestral haplotype. Hap-2, Hap-3, Hap-4, Hap-5, and Hap-6 are connected to Hap-1, with each haplotype exhibiting varying degrees of divergence.

#### 3.2.3. Correlation Analysis of Haplotype Diversity and Environmental Factors

**Figure 6:**
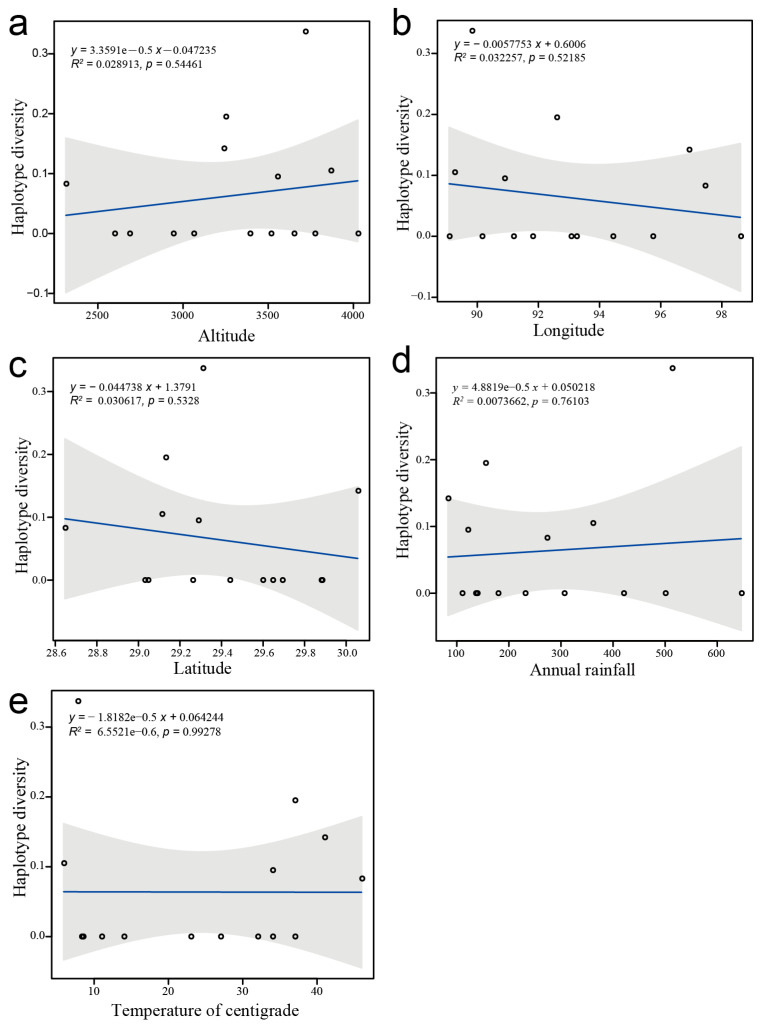
Correlation between haplotype diversity (Hd) and five environmental factors across 15 *D. stramonium* populations in Tibet, China. The blue line represents the linear regression trend, and the grey shaded area denotes the 95% confidence interval. (**a**) altitude; (**b**) longitude; (**c**) latitude; (**d**) annual rainfall; (**e**) temperature. Statistical analyses indicated no significant correlations between Hd and any of the environmental variables (*p* > 0.05).

The results of the correlation analysis between haplotype diversity index (Hd) and environmental factors are presented in Figure 6. No significant correlation was found between haplotype diversity index (Hd) and altitude, longitude, latitude, annual rainfall, and temperature among the 15 *D. stramonium* populations.

### 3.3. Niche Characteristics of D. stramonium

#### 3.3.1. Community-Dominant Species Importance Values and Niche Breadth

A total of 61 species, representing 20 families and 49 genera, were recorded in the community structure survey (Table A1). Species with an importance value greater than 2 were identified as dominant species. The importance values and niche breadths of these dominant species are provided in Table 2. *D. stramonium* exhibited the highest importance value at 21.92, followed by *Dysphania schraderiana* (8.59), *Chenopodium album* (8.19), *Galinsoga parviflora* (4.94), *Tribulus terrestris* (4.36), and *Amaranthus hybridus* (4.02). Among these, *D. stramonium* also exhibited the highest niche breadth.

#### 3.3.2. Niche Overlap Among Community-Dominant Species

The niche overlap among the 16 dominant species investigated in this survey is shown in Table A2. Two species pairs, *Malva pusilla*-*Malva verticillata* var. *rafiqii* and *Malva pusilla*-*Salsola collina*, had overlap values between [0, 0.100]. There were 44 and 74 pairs of dominant species with overlap between [0.100, 0.500] and [0.500, 1], respectively, indicating that niche overlap among dominant species was common. The top three species pairs with the highest niche overlap were *Dysphania schraderiana*–*C. album* (0.867), *D. stramonium*–*C. album* (0.849), and *D. stramonium*–*Dysphania schraderiana* (0.845), suggesting intense competition among these species.

#### 3.3.3. Correlation Analysis Between Community Species Diversity Index and Invasion Intensity Index

**Figure 7:**
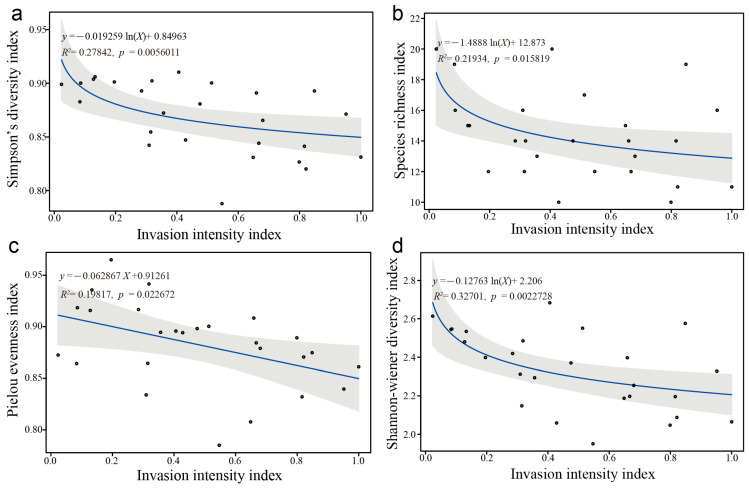
Correlations between *D*. *stramonium* invasion intensity and four community biodiversity indices. (**a**) Simpson’s diversity index, (**b**) species richness index, (**c**) Pielou’s evenness index, and (**d**) Shannon–Wiener diversity index.

The correlation analysis results between *D. stramonium* invasion intensity index and various diversity indices, including the Simpson diversity index, Species richness index, Pielou evenness index, and Shannon–Wiener diversity index, are depicted in Figure 7. The data indicate a significant negative correlation, where increasing intensity of *D. stramonium* invasion corresponds with decreasing species diversity within the plant community.

## 4. Discussion

### 4.1. Mechanistic Interpretation of D. stramonium Allelopathy

Invasive plants often interfere with native species through the release of allelopathic compounds, which can suppress seed germination, seedling establishment, and competitive performance of neighboring plants, thereby enhancing their own ecological advantage [25]. The strength of allelopathic inhibition typically depends on both the plant organ in which allelochemicals are synthesized and accumulated [26] and the concentration of these substances [27]. Previous studies have shown that *D. stramonium* contains several bioactive allelopathic compounds, particularly tropane alkaloids such as atropine, scopolamine, and related derivatives, which have been reported to inhibit germination and early seedling growth in other plant species (e.g., Solanaceae weeds and cereals) [28,29].

Our results demonstrate that aqueous extracts of *D. stramonium* roots, stems, and leaves exerted significant inhibitory effects on the germination of *H. vulgare* var. *coeleste* and *P. sativum*. However, the inhibitory intensity varied among plant organs and between receptor species (Figure 2). For *H. vulgare* var. *coeleste*, the strongest inhibition consistently came from leaf extracts across all concentrations, followed by stems and roots, suggesting a higher accumulation or release efficiency of allelopathic compounds in leaves. In *P. sativum*, stem extracts showed the strongest inhibition, with root and leaf extracts following, and the inhibitory pattern shifted at higher concentrations (0.075–0.1 g/mL), reflecting concentration-dependent and species-specific sensitivity.

The non-linear responses observed in both species indicate that different germination parameters (germination rate, potential, and index) responded unequally to extract concentration, influencing the comprehensive inhibition values. Collectively, these findings highlight that the allelopathic effects of *D. stramonium* are shaped by plant organ, extract concentration, and receptor species, and may be attributed to the differential distribution of tropane alkaloids or other allelochemicals within plant tissues.

### 4.2. Population Differentiation, Dynamics, and Haplotype Distribution in D. stramonium

#### 4.2.1. Genetic Differentiation in *D. stramonium* Populations

Genetic differentiation among the *D. stramonium* populations in Tibet is low, as indicated by the overall Fst value of 0.05871. Such weak genetic structure is consistent with the general patterns observed in many invasive annual plant species, which often exhibit shallow population differentiation due to recent introductions, rapid range expansion, and insufficient time for strong spatial divergence to accumulate [30]. The low Nst and Gst values observed in this study, together with the pattern of Nst < Gst, indicate the absence of a clear phylogeographic structure among Tibetan populations, suggesting that haplotype distributions are not strongly shaped by geographic distance. Similar patterns of weak regional genetic structures have been reported in other rapidly spreading invasive herbs [31]. Although *D. stramonium* was introduced to China several centuries ago, historical records and field surveys indicate that its spread into Tibet is much more recent—likely within the past few decades. Therefore, the low genetic differentiation and demographic signatures detected here are consistent with a recent regional expansion of the species within Tibet.

#### 4.2.2. Historical Population Dynamics of *D. stramonium*

Tajima’s D-value and mismatch distribution curves are commonly used to analyze population dynamics and detect historical population expansions [32,33]. In this study, Tajima’s D-value for the 15 *D. stramonium* populations in Tibet was −1.76007, which is significant at the *p* < 0.05 level, suggesting that these populations in Tibet do not follow neutral evolutionary theory and have historically undergone population expansion [34]. The mismatch distribution analysis showed a close fit between the observed and predicted values, with a single main peak and an overall downward trend in the curve (Figure 3). These results are consistent with those of the neutrality test and indicate recent population expansion in *D. stramonium* populations in Tibet.

#### 4.2.3. Interpretation of Haplotype Patterns in *D. stramonium*

Our study identified 6 haplotypes among the 15 *D. stramonium* populations in Tibet. Visualizing the distribution of individuals within each haplotype revealed that most of the individuals belong to original haplotypes, with frequent gene exchanges among populations (Figure 5). This suggests that *D. stramonium* populations in Tibet have not significantly differentiated new haplotypes, with only a few individuals exhibiting genetic mutations leading to new haplotype types. The index of differentiation between populations, (Nst < Gst) indicates that the distribution of *D. stramonium* in Tibet lacks a clear geographic structure [35]. Furthermore, the haplotype network map and geographic distribution analyses showed that the six haplotypes did not form an evident group, and haplotypes within each geographic population were scattered, further confirming the lack of a geographic structure according to their geographic origins (Figure 4). This aligns with the results of the computation of the inter-habitat differentiation indices (Nst and Gst). The correlation analysis between haplotype diversity index (Hd) and environmental factors, including altitude, latitude, longitude, mean annual temperature, and mean annual rainfall, showed no significant correlation (Figure 6). Field surveys and communications with local residents of the sampling sites suggest that the invasion of *D. stramonium* in Tibet is likely driven by anthropogenic introductions, as the plant is often cultivated as an ornamental flower in the region.

### 4.3. Niche Characteristics of Dominant Species in D. stramonium Communities

#### 4.3.1. Dominant Species Importance Values and Niche Breadth

Niche breadth reflects a species’ ability to adapt to its environment and utilize resources [36], which in turn can indicate its functional status within its biotope [37]. Generally, a species with a greater niche breadth has a higher adaptability to environmental factors and tends to occupy a dominant position within the community owing to greater competitiveness of the species. In this study, *D. stramonium* had the largest niche breadth, with Shannon–Wiener and Levins niche breadth values of 3.198 and 23.326, respectively, with the values of dominant species in the plant community being generally consistent. These results indicate that *D. stramonium* has the strongest ability to adapt to environmental factors such as light and water, efficiently utilizes community resources, and has the most extensive distribution in the community. Moreover, it has achieved a dominant status within the whole plant community and is a crucial component for the construction of the plant community.

#### 4.3.2. Niche Overlap Among Dominant Species

Niche overlap measures the extent to which species share environmental resources, reflecting the competitive relationships between species with similar niches [38]. In general, species with larger niche breadths tend to have greater niche overlap with other species, indicating a higher level of competition for resources [39]. In this study, of 120 dominant species pairs, the highest niche overlap was observed among the dominant species pairs *D. stramonium*–*Dysphania schraderiana*, *D. stramonium*–*C. album*, and *Dysphania schraderiana*–*C. album*. These species also had the highest niche breadth values, suggesting that they share a wide range of environmental resources and prefer similar habitats such as roadsides, fields, and areas near houses. In addition, significant niche overlap was observed between species with smaller niche breadths, such as *Malva pusilla*–*Medicago lupulina* (0.533) and *Malva pusilla*–*Echinochloa crus-galli* (0.627), likely due to the limited living space and scarcity of environmental resources in their habitats (such as wasteland, construction waste land, and roadsides). Conversely, the niche overlap between *Malva pusilla*–*S. collina* (0.080) and *Malva pusilla*–*Malva verticillata* var. *rafiqii* (0.081) was low, possibly due to the differences in their ecological and biological characteristics. These differences result in cross-distribution among resource niches, where the usage of environmental resources and the need for overlap are minimal, indicating a complementary rather than a competitive relationship. The plant invasion intensity index, which indicates the extent of invasion within a plant community, showed a significant negative correlation with four community biodiversity indices, suggesting that *D. stramonium* invasion severely reduces native plant diversity (Figure 7).

## 5. Conclusions

As an invasive plant, *D. stramonium* effectively inhibits seed germination through allelopathic effects in invaded sites. Compared to native plants, this species demonstrates a greater ability to adapt to the environment and compete for environmental resources, which gives it a competitive edge in community establishment. Additionally, *D. stramonium* tends to spread and proliferate widely in urban areas, often due to anthropogenic introduction. We hypothesize that the successful invasion of *D. stramonium* in Tibet is attributed to its strong allelopathic effects, high ecological adaptability, competitive resource acquisition, and anthropogenic introduction. The invasion of *D. stramonium* significantly impacts the diversity of native plant species, making the development of scientific management strategies crucial for protecting plant diversity in affected areas.

## Figures and Tables

**Table 1:** Sampling information of *D. stramonium* populations collected in Tibet, China.

Location	Code	Longitude (°E)	Latitude (°N)	Individuals Collected	Voucher Number
Lhasa City, Tibet	LSS	91.18103	29.64584	26	Wjw20230701
Gongga County, Tibet	GGX	90.88770	29.28778	21	Wjw20230702
Nimu County, Tibet	NMX	90.15169	29.43933	23	Wjw20230703
Renbu County, Tibet	RBX	89.82179	29.30988	20	Wjw20230704
Bailang County, Tibet	BLX	89.25534	29.11256	19	Wjw20230705
Nanmulin County, Tibet	NML	89.07829	29.69206	23	Wjw20230706
Shannan City, Tibet	SNS	91.80690	29.26065	24	Wjw20230707
Jiacha County, Tibet	JCX	92.59348	29.13084	20	Wjw20230708
Lang County, Tibet	LX	93.06258	29.04554	22	Wjw20230709
Gongbujiangda County, Tibet	GBJD	93.24185	29.88319	23	Wjw20230710
Linzhi City, Tibet	LZS	94.43109	29.59796	20	Wjw20230711
Bomi County, Tibet	BMX	95.73593	29.87905	24	Wjw20230712
Basu County, Tibet	BSX	96.92151	30.05609	27	Wjw20230713
Mangkang County, Tibet	MKX	98.60788	29.03024	20	Wjw20230714
Chayu County, Tibet	CYX	97.44478	28.64630	24	Wjw20230715

**Table 2:** Importance value and niche breadth of community-dominant species.

Species	Importance Value	Niche Breadth	
		Shannon-Wiener	Levins
*Datura stramonium*	21.92	3.198	23.326
*Dysphania schraderiana*	8.59	3.163	22.329
*Chenopodium album*	8.19	3.164	22.292
*Galinsoga parviflora*	4.94	2.856	15.419
*Tribulus terrestris*	4.36	2.78	15.238
*Amaranthus hybridus*	4.02	2.842	16.143
*Chloris virgata*	3.73	2.179	14.331
*Eragrostis nigra*	3.61	2.868	17.226
*Salsola collina*	3.21	2.476	10.784
*Artemisia sieversiana*	2.69	2.673	13.843
*Pennisetum flaccidum*	2.65	2.609	13.165
*Malva verticillata*	2.61	2.549	12.595
*Lepidium apetalum*	2.41	2.221	8.501
*Echinochloa crus-galli*	2.25	2.462	11.422
*Medicago lupulina*	2.19	2.473	11.708
*Malva pusilla*	2.16	2.262	9.255

## Data Availability

All links to input data are reported in the manuscript and all output data are available upon request to the authors.

## Appendix A

**Table A1:** List of plants surveyed for the *D. stramonium* community.

Family	Genus	Species
Amaranthaceae	*Amaranthus*	*Amaranthus hybridus*
	*Oxybasis*	*Oxybasis glauca*
Araceae	*Arisaema*	*Arisaema flavum*
Asteraceae	*Galinsoga*	*Galinsoga parviflora*
	*Sonchus*	*Sonchus oleraceus*
	*Artemisia*	*Artemisia sieversiana*
	*Cosmos*	*Cosmos bipinnatus*
	*Senecio*	*Senecio vulgaris*
	*Cirsium*	*Cirsium arvense* var. *alpestre*
	*Cirsium*	*Cirsium arvense*
	*Gnaphalium*	*Pseudognaphalium affine*
	*Taraxacum*	*Taraxacum mongolicum*
	*Artemisia*	*Artemisia demissa*
	*Artemisia*	*Artemisia wellbyi*
Boraginaceae	*Cynoglossum*	*Cynoglossum amabile*
Chenopodiaceae	*Chenopodium*	*Dysphania schraderiana*
	*Salsola*	*Kali collinum*
	*Chenopodium*	*Chenopodium album*
Convolvulaceae	*Pharbitis*	*Ipomoea purpurea*
	*Convolvulus*	*Convolvulus arvensis*
Cruciferae	*Brassica*	*Brassica napus*
	*Rorippa*	*Rorippa palustris*
	*Lepidium*	*Lepidium apetalum*
	*Capsella*	*Capsella bursa-pastoris*
	*Descurainia*	*Descurainia sophia*
	*Brassica*	*Brassica rapa*
Geraniaceae	*Erodium*	*Erodium cicutarium*
Gramineae	*Echinochloa*	*Echinochloa crus-galli*
	*Pennisetum*	*Pennisetum flaccidum*
	*Setaria*	*Setaria viridis*
	*Poa*	*Poa annua*
	*Chloris*	*Chloris virgata*
	*Eragrostis*	*Eragrostis nigra*
	*Digitaria*	*Digitaria cruciata*
	*Bothriochloa*	*Bothriochloa ischaemum*
	*Avena*	*Avena fatua*
	*Elymus*	*Elymus tangutorum*
Labiatae	*Elsholtzia*	*Elsholtzia densa*
Leguminosae	*Sophora*	*Sophora moorcroftiana*
	*Medicago*	*Medicago edgeworthii*
	*Medicago*	*Medicago lupulina*
	*Melilotus*	*Melilotus suaveolens*
	*Astragalus*	*Astragalus strictus*
Loganiaceae	*Buddleja*	*Buddleja alternifolia*
Malvaceae	*Malva*	*Malva pusilla*
	*Malva*	*Malva verticillata*
Plantaginaceae	*Plantago*	*Plantago depressa*
Polygonaceae	*Fagopyrum*	*Fagopyrum tataricum*
	*Persicaria*	*Persicaria nepalensis*
	*Rumex*	*Rumex nepalensis*
	*Persicaria*	*Polygonum aviculare*
	*Persicaria*	*Persicaria hydropiper*
	*Persicaria*	*Persicaria lapathifolia*
	*Fagopyrum*	*Fagopyrum esculentum*
Portulacaceae	*Portulaca*	*Portulaca oleracea*
Rubiaceae	*Galium*	*Galium spurium*
Solanaceae	*Datura*	*D. stramonium*
	*Nicandra*	*Nicandra physalodes*
	*Datura*	*Datura metel*
Tamaricaceae	*Myricaria*	*Myricaria wardii*
Zygophyllaceae	*Tribulus*	*Tribulus terrestris*

**Table A2:** Niche overlap of dominant shrub species.

Species	S1	S2	S3	S4	S5	S6	S7	S8	S9	S10	S11	S12	S13	S14	S15	S16
S1	1															
S2	0.845	1														
S3	0.849	0.867	1													
S4	0.621	0.696	0.741	1												
S5	0.698	0.701	0.622	0.504	1											
S6	0.779	0.69	0.694	0.59	0.562	1										
S7	0.722	0.672	0.695	0.411	0.593	0.489	1									
S8	0.824	0.707	0.694	0.456	0.78	0.503	0.778	1								
S9	0.582	0.685	0.502	0.379	0.536	0.487	0.439	0.573	1							
S10	0.586	0.67	0.724	0.754	0.551	0.532	0.436	0.475	0.538	1						
S11	0.591	0.667	0.679	0.647	0.424	0.554	0.626	0.578	0.512	0.478	1					
S12	0.694	0.645	0.695	0.369	0.432	0.421	0.698	0.598	0.553	0.438	0.498	1				
S13	0.529	0.483	0.445	0.285	0.691	0.292	0.534	0.699	0.391	0.242	0.36	0.463	1			
S14	0.64	0.553	0.574	0.662	0.49	0.594	0.399	0.344	0.232	0.573	0.255	0.32	0.177	1		
S15	0.658	0.553	0.738	0.546	0.45	0.625	0.363	0.461	0.329	0.471	0.41	0.461	0.244	0.389	1	
S16	0.51	0.511	0.557	0.744	0.429	0.627	0.11	0.327	0.08	0.506	0.443	0.081	0.108	0.627	0.533	1

Note: S1–S16 denote species D. stramonium, D. schraderiana, C. album, G. parviflora, T. terrestris, A. hybridus, C. virgata, E. nigra, S. collina, A. sieversiana, P. flaccidum, M. verticillata, L. apetalum, E. crus-galli, M. lupulina, M. pusilla.
